# Associations of breast cancer related exposures and gene expression profiles in normal breast tissue—The Norwegian Women and Cancer normal breast tissue study

**DOI:** 10.1002/cnr2.1777

**Published:** 2023-01-08

**Authors:** Sanda Krum‐Hansen, Karina Standahl Olsen, Endre Anderssen, Jan Ole Frantzen, Eiliv Lund, Ruth H. Paulssen

**Affiliations:** ^1^ Department of Community Medicine UiT The Arctic University of Norway Tromsø Norway; ^2^ Department of Hematology and Oncology Stavanger University Hospital Stavanger Norway; ^3^ Genomics Support Center Tromsø (GSCT) UiT The Arctic University of Norway Tromsø Norway; ^4^ Narvik Hospital University Hospital of North Norway Narvik Norway; ^5^ Department of Clinical Medicine UiT The Arctic University of Norway Tromsø Norway

**Keywords:** alcohol consumption, breast cancer, breast tissue, gene expression, hormone therapy, microarray, normal tissue, obesity, parity, smoking

## Abstract

**Background:**

Normal breast tissue is utilized in tissue‐based studies of breast carcinogenesis. While gene expression in breast tumor tissue is well explored, our knowledge of transcriptomic signatures in normal breast tissue is still incomplete. The aim of this study was to investigate variability of gene expression in a large sample of normal breast tissue biopsies, according to breast cancer related exposures (obesity, smoking, alcohol, hormone therapy, and parity).

**Methods:**

We analyzed gene expression profiles from 311 normal breast tissue biopsies from cancer‐free, post‐menopausal women, using Illumina bead chip arrays. Principal component analysis and *K*‐means clustering was used for initial analysis of the dataset. The association of exposures and covariates with gene expression was determined using linear models for microarrays.

**Results:**

Heterogeneity of the breast tissue and cell composition had the strongest influence on gene expression profiles. After adjusting for cell composition, obesity, smoking, and alcohol showed the highest numbers of associated genes and pathways, whereas hormone therapy and parity were associated with negligible gene expression differences.

**Conclusion:**

Our results provide insight into associations between major exposures and gene expression profiles and provide an informative baseline for improved understanding of exposure‐related molecular events in normal breast tissue of cancer‐free, post‐menopausal women.

## 1 BACKGROUND

Breast cancer is now the most frequently diagnosed cancer worldwide, with more than two million new cases per year.^1^ The main risk factors for breast cancer, other than sex and age, include overweight/obesity, alcohol consumption, family history of breast cancer, reproductive history, postmenopausal hormone therapy (HT), as well as smoking for pre‐menopausal breast cancer.^2^, ^3^ With growing incidence rates, increased understanding of the mechanisms of cancer development is needed for preventive measures and early detection.

Despite growing understanding of breast cancer development at the molecular level, our knowledge of transcriptomic signatures of normal breast tissue is still incomplete. Tissue samples of normal breast have been widely used in breast cancer research serving as control tissue.^4^, ^5^, ^6^, ^7^ However, these tissue samples often originate from reduction mammoplasty, benign breast lesions, or histologically normal tissue adjacent to breast cancer.^4^, ^5^, ^8^ Such tissue samples often show more histological abnormalities when compared to tissue obtained from healthy donors,^9^, ^10^, ^11^ and using different sources of control breast tissue in different studies make comparisons between studies difficult. In addition, most studies on gene expression profiles were generated from a small set of samples that were likely not representative of the general population.^8^, ^12^ Finally, a better understanding of the natural variability of gene expression in normal breast tissue would represent a significant step forward in our understanding of early disease‐related mechanisms.

With this study of a large, random sample of cancer‐free, post‐menopausal Norwegian women, we investigated the variability of gene expression in normal breast tissue. In particular, we explored gene expression patterns associated with exposure to known risk factors for breast cancer, such as obesity, parity, alcohol consumption, and use of menopausal HT. We also examined if smoking was associated with gene expression. The generated data represent a baseline of gene expression patterns in normal breast tissue from cancer‐free, post‐menopausal women, and can potentially play an important role in the feasibility, design, and analysis of future tissue‐based studies investigating biomarkers of exposure, as well as breast cancer development.

## 2 MATERIAL AND METHODS

### 2.1 Study population

A detailed description of the recruitment proses and study population as well as ethical aspects of genetic research in healthy populations are presented and discussed in our previously published article.^13^ Briefly, study participants were recruited through the national mammography screening program at the Breast Diagnostic Center at the University Hospital of North Norway (UNN), Tromsø, Norway, during the years 2010–2011. They were not referred due to pathological clinical findings or irregularities on previous mammograms but attended a scheduled routine mammogram. Eligible women were age between 53 and 67 years, were post‐menopausal, and were already participating in the nationally representative Norwegian Women and Cancer study (NOWAC).^14^ Exclusion criteria for the present study included self‐reported previous history of breast cancer, positive mammogram, other current malignant diseases and use of anticoagulation therapy with warfarin, heparin, dipyridamole, or clopidogrel. Eligible women who agreed to participate received written and oral information, signed an informed consent form, and answered a two‐page questionnaire regarding menopausal status, weight and height, smoking and alcohol consumption, use of HT and other medication. The number of included participants was 317. Three years after inclusion, data was linked to the Cancer Registry of Norway, using the unique personal identification number. This resulted in the exclusion of five participants who developed breast cancer within 3 years after the biopsy was taken, and one participant due to prior lymphoma diagnosis with unknown treatment. Thus, the final number of women included for statistical analysis was 311. The North Norway Regional Committee for Medical and Health Research Ethics (REK‐Nord case no # 200603551) approved the study.

### 2.2 Definition of exposures

Information on year of birth, menopausal status, current height and weight and exposures (HT use, smoking and alcohol consumption) was extracted from the two‐page questionnaires answered at the time of inclusion.

Body mass index (BMI) was calculated, and obesity was defined according to the definition of the World Health Organization (WHO, BMI > 30). Women were considered post‐menopausal if they reported that menstruation had ceased. In case of incomplete information, women were defined as post‐menopausal if they were older than 53 years. Women who had consumed alcohol during the week prior to the biopsy, regardless of the type or amount, were defined as alcohol consumers. Similarly, women who had smoked during the week prior to biopsy were defined as smokers. Only women who were current users of systemic HT (tablets or patch) were defined as HT users. Data on parity was retrieved from the NOWAC database, and the variable was dichotomized into parous versus non‐parous for analyses of gene expression.

We also collected data on smoking status and alcohol consumption (g/day) from the more comprehensive eight‐page questionnaire answered by the participants as part of the prospective data collection in NOWAC. Participants of the biopsy study answered the eight‐page questionnaire 0–20 years prior to donating a biopsy (1991–2011). These data were used for a sensitivity analysis.

### 2.3 Tissue samples

An experienced radiologist obtained tissue samples after mammography, by ultrasound‐guided needle‐biopsy (14 gauges) from the gland tissue of the upper lateral quadrant of the left breast. The procedure was standardized. We collected one biopsy from every participant. In case of macroscopically sparse material, a second biopsy was obtained. The biopsies were kept at room temperature in RNA Later (Qiagen) for <24 h until storage at −70°C.

### 2.4 RNA preparation

RNA preparation was conducted at The Department of Cancer Genetics, Oslo University Hospital, Oslo, Norway. Tissue biopsies were homogenized using TissueLyser LT (Qiagen, Hilden, Germany) and 5 mm steel beads. Total RNA and genomic DNA were isolated using the AllPrep DNA/RNA Mini Kit from Qiagen (Cat. No. 80204) and the QIAcube instrument (Qiagen, Hilden, Germany). A custom protocol was followed for the extraction (RNA_AllPrepDNARNA_AnimalCells_AllPrep350_ID2481). RNA was stored at −80°C. RNA quantity was measured by NanoDrop (Thermo Fisher Scientific, Wilmington, Delaware, USA). The BioAnalyzer 2100 and Agilent RNA 6000 Nano kit (cat. No. 5067–1511) were used to evaluate RNA integrity (Agilent, Santa Clara, US).

### 2.5 Gene expression analysis

mRNA gene expression was analyzed at a certified Illumina service provider (NTNU Genomics Core Facility, Trondheim, Norway). Briefly, RNA was amplified with Ambion's Illumina® TotalPrep RNA amplification kit (Cat #AMIL 1791) using 400 ng of total RNA as input material. Incorporation of biotin‐labeled nucleotides was performed overnight (14 h) at 37°C in vitro transcription (IVT). cRNA was quantified using the NanoDrop ND‐1000 (NanoDrop, Wilmington, USA), and cRNA integrity was determined by electrophoresis using the Experion Bioanalyzer (BioRad). A total of 750 ng of biotin‐labeled cRNA was hybridized to IlluminaHumanHT‐12 v.4 expression bead chip (Illumina®). Beadchips were scanned with Illumina BeadArray Reader. Numerical results were extracted with Bead Studio v3.0.19.0 without any normalization or background subtraction.

### 2.6 Statistical analysis

Data analysis was done using R (r-project.org). Raw files were quantile normalized using the Bioconductor lumi package.^15^ Principal component analysis (PCA) and clustering was used for initial analysis of the dataset. The PCA was computed with all genes included. In order to obtain distinct clusters that correlate with the PCA scores to simplify interpretation we clustered the genes with the most variability (inter quantile range [IQR] > 1 log2 unit).

The three gene clusters identified were analyzed for overrepresented gene ontology (GO) terms using the clusterProfiler package.^16^ This analysis highlighted cell composition as a potentially important co variate and non‐negative matrix factorization (NMF) was used to obtain improved cellular composition estimates.^17^ NMF was run with the “nsNMF” method and initialized with non‐negative singular value decomposition to get a sparser estimate for the gene profiles of the cell types. The association of exposures and covariates on gene expression was determined using linear models for microarrays (LIMMA), with the scores on principal component one and two included as covariates to correct for bias due to the cellular composition of the biopsies. *p* Values from the linear models were corrected for multiple testing using the method of Benjamin and Hochberg.^18^ Finally Camera^19^ was used to identify pathways and GO terms that were related to the exposure variables. The Camera analysis was carried out with all genes using the same model as for the limma analysis, that is, PC1 and PC2 were included to correct for cell type composition.

STATA (StataCorp. 2017. Stata Statistical Software: Release 15. College Station, TX: StataCorp LLC.) was used for descriptive statistics. *T* tests were used for BMI and age as continuous variables, and chi‐square tests were used for the categorical variables.

## 3 RESULTS

### 3.1 Characteristics of study participants

For this study, 311 cancer‐free, post‐menopausal women were included. Average age of the study population was 60 years. More than half of the participants (54.7%) were classified as overweight according to WHO (BMI > 25), with an average BMI for the whole study populations of 26.2. More than 79% of the women had consumed alcohol, and 21% had been smoking prior to biopsy sampling. Very few participants used HT (8.7%), and most of the women had completed at least one full term pregnancy (Table 1).

**TABLE 1:** Descriptive characteristics of study population (*n* = 311)

		*n* (%)	Age, mean	BMI, mean	Current smokers, *n* (%)	Alcohol users, *n* (%)	HT users, *n* (%)	Parous, yes (%)	*n* Children, mean
Total		311	60.0	26.2	66 (21.2)	241 (79.0)	27 (8.7)	256 (82.3)	2.1
Obese	No (BMI < 30)	261 (83.9)	60.0	24.8	52 (19.9)	208 (80.9)	25 (9.6)	219 (83.9)	2.1
	Yes (BMI > 30)	50 (16.0)	60.7	33.4	14 (28)	33 (68.8)	2 (4.0)	37 (74.0)	2.2
	*p*, <30 vs. >30		.20		.20	.06	.27	.09	.60
Current smokers	No	245 (78.7)	60.3	26.1		195 (81.2)	25 (10.2)	201 (82.0)	2
	Yes	66 (21.2)	59.3	26.9		46 (70.7)	2 (3.0)	55 (83.3)	2.2
	*p*, no vs. yes		.07	.32		.07	.08	.80	.16
Alcohol users	No	64 (20.5)	61.2	27.4	19 (29.9)		5 (7.8)	53 (82.8)	2.2
	Yes	241 (77.5)	59.8	25.9	46 (19.0)		21 (8.7)	199 (82.6)	2.1
	*p*, no vs. yes		.01	.02	.06		.80	.96	.60
HT users	No	283 (90.9)	60.1	26.3	64 (22.6)	220 (78.8)		237 (83.7)	2.2
	Yes	27 (8.7)	59.6	25.7	2 (7.4)	21 (80.7)		18 (66.6)	1.6
	*p*, no vs. yes		.55	.33	.08	.80		.03	.04
Parous	No	55 (17.7)	60.4	26.7	11 (20.0)	42 (79.2)	9 (16.4)		
	Yes	256 (82.3)	60.0	26.11	55 (21.5)	199 (78.9)	18 (7.0)		2.3
	*p*, no vs. yes		.45	.34	.80	.90	.03		

Abbreviations: BMI, body mass index; HT, hormone therapy.

### 3.2 Gene expression in normal breast tissue

#### 3.2.1 Unsupervised clustering

**FIGURE 1:**
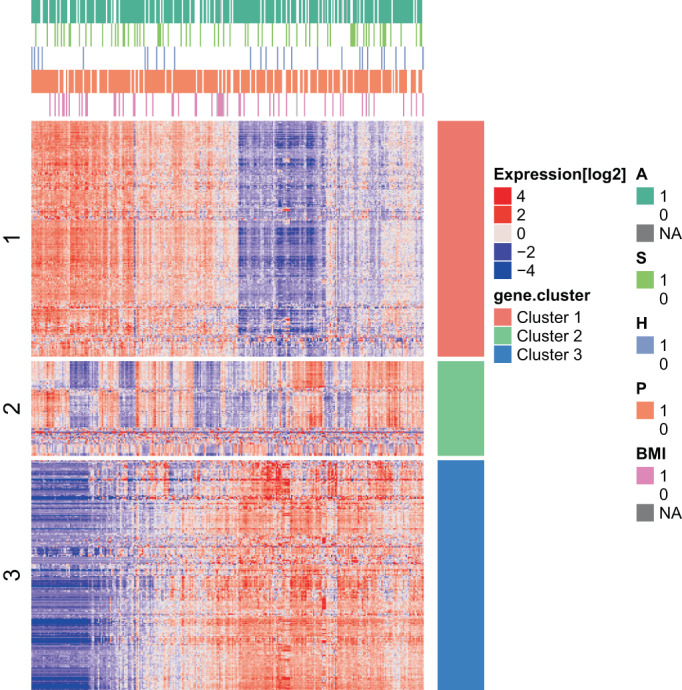
High‐variance genes in breast tissue from healthy women represent three main clusters unrelated to major breast cancer risk factors. We analyzed 607 high‐variance genes (inter quantile range larger than 1 log2 unit) by *K*‐means clustering, and identified three dominating clusters (1‐red, 2‐green, and 3‐blue). Distribution of the exposures are shown in the top pane (in color, legend to the right). A, alcohol; BMI, body mass index; H, HT use; P, parity; S, smoking

After normalization of data, the initial analyses identified 607 genes with high level of variance with IQR larger than one log2 unit. These 607 genes were analyzed by *K*‐means clustering and three dominating cluster were identified (Figure 1). These clusters appear unrelated to either of the exposures. PCA analysis with the exposure variables illustrated are shown in Figure S1.

**FIGURE 2:**
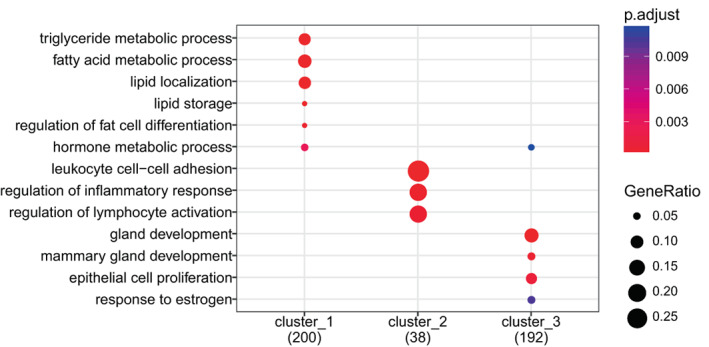
The three main gene expression clusters identified in breast tissue from healthy women likely reflect biopsy composition. Genes from the three clusters identified using *K*‐means clustering (Figure 1) were analyzed using clusterProfiler, to identify overrepresented gene ontology categories that describe the functionality of the clusters

Genes from the three clusters were analyzed using cluster profiler to identify GO categories that describe the functionality of the clusters (Figure 2).

Cluster 1 is dominated by processes related to epithelial cells, both differentiation processes, cell functions, and proliferation. Cluster 2 is dominated by genes involved in immune system processes and immune cell specific processes such as leucocyte chemotaxis, regulation of leucocytes proliferation, and interleukin production. Metabolic genes specifically related to lipid metabolism and fat cell differentiation dominate the third cluster.

**FIGURE 3:**
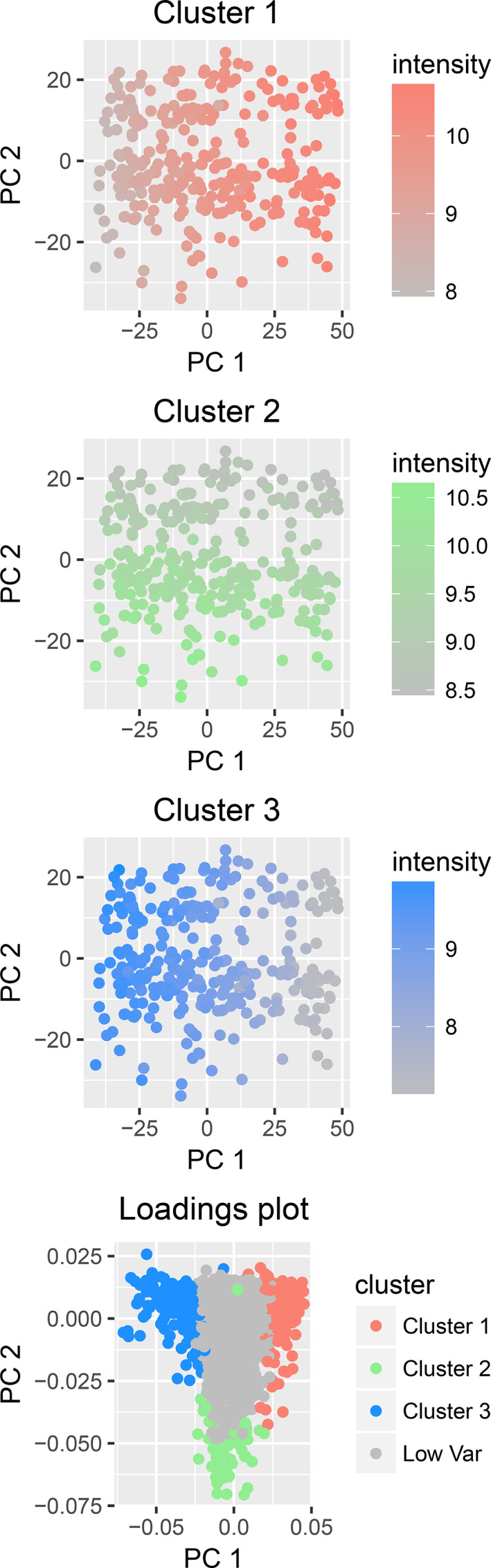
The total variability in gene expression data from breast tissue from healthy women is dominated by the balance of fatty tissue and epithelial tissue. Principal component analysis of the gene expression data was performed. Based on the overrepresented gene ontology terms of each cluster (Figure 2), the principal components of the loadings plot reflect biopsy composition represented by cluster 1 (fatty tissue), cluster 2 (immune cells), and cluster 3 (epithelial tissue)

PCA of the full dataset showed that 47% of the total variability of the gene expression data was captured by the two first principal components. Principal component one therefore reflects the balance between fatty tissue and epithelial tissue in the biopsy and principal component two reflects the fraction of immune cells included (Figure 3). NMF identified three factors that correlate with well‐known cell type markers (Supporting Information S1). However, as the dominating cell types are epithelial and adipose cells with only a small fraction of immune cells, epithelial and adipose markers correlate strongly (negatively and positively respectively) with the first principal component. This enables the use of a single factor (PC1) to represent epithelial versus adipose tissue which reduces collinearity problems in the linear modeling. Hence, NMF was not included in further analyses.

#### 3.2.2 Associations between exposures and gene expression profiles

To analyze associations of selected exposures with gene expression profiles, we used LIMMA. An overview of the results is presented in Table 2. The 20 most significant genes and pathways associated with each exposure are presented in Tables 3 and 4 for obesity, Tables 5 and 6 for smoking, Tables 7 and 8 for alcohol, Tables 9 and 10 for HT, and Tables 11 and 12 for parity. The list of differentially expressed genes and gene sets are provided in Supporting Information S2 and S3.

**TABLE 2:** Overview of results: Genes and pathways associated with exposures

Exposure	Single genes (*n*)			Pathways (*n*)		
	Total	Upreg.	Downreg	Total	Upreg.	Downreg.
Obesity	1577	812	765	606	527	79
Smoking	10	9	1	19	19	0
Alcohol	9	8	1	80	50	30
HT	2	2	0	0	0	0
Parity	0	0	0	0	0	0

Abbreviations: downreg., downregulated; HT, hormone therapy; upreg., upregulated.

**TABLE 3:** Top 20 genes associated with obesity

Entrez ID	Gene symbol	Fold change	Adj. *p* value
124	ADH1A	−0.4839	<.001
125	ADH1B	−0.4241	<.001
137872	ADHFE1	−0.2960	<.001
122622	ADSSL1	−0.2956	<.001
283	ANG	−0.2377	<.001
398	ARHGDIG	0.1761	<.001
618	BCYRN1	0.5317	<.001
80763	SPX	−0.5217	<.001
78995	C17orf53	−0.1484	<.001
717	C2	0.2267	<.001
54976	C20orf27	−0.2612	<.001
719	C3AR1	0.1931	<.001
728	C5AR1	0.2035	<.001
85027	SMIM3	−0.1984	<.001
154791	C7orf55	−0.2249	<.001
56997	ADCK3	−0.3315	<.001
1230	CCR1	0.1737	<.001
8832	CD84	0.1881	<.001
1066	CES1	0.6174	<.001
1149	CIDEA	−0.6599	<.001

**TABLE 4:** Top 20 gene sets associated with obesity

Pathway ID	Number of genes	Direction	Adj. *p* value	Category name
GO:0033108	73	Down	3.10E‐13	Mitochondrial respiratory chain complex assembly
GO:0010257	56	Down	7.23E‐12	NADH dehydrogenase complex assembly
GO:0032981	56	Down	7.23E‐12	Mitochondrial respiratory chain complex I assembly
GO:0097031	56	Down	7.23E‐12	Mitochondrial respiratory chain complex I biogenesis
GO:0022904	126	Down	1.40E‐11	Respiratory electron transport chain
GO:0022900	128	Down	3.81E‐11	Electron transport chain
GO:0045333	193	Down	1.12E‐10	Cellular respiration
GO:0042775	62	Down	2.48E‐09	Mitochondrial ATP synthesis coupled electron transport
GO:0042773	63	Down	3.81E‐09	ATP synthesis coupled electron transport
GO:0006415	178	Down	5.15E‐09	Translational termination
GO:0070124	84	Down	5.90E‐09	Mitochondrial translational initiation
GO:0070125	84	Down	7.03E‐09	Mitochondrial translational elongation
GO:0070126	86	Down	9.14E‐09	Mitochondrial translational termination
hsa00190	119	Down	1.36E‐08	Oxidative phosphorylation
GO:0006119	84	Down	1.80E‐08	Oxidative phosphorylation
GO:0032543	117	Down	6.16E‐08	Mitochondrial translation
GO:0006414	207	Down	6.45E‐08	Translational elongation
hsa05012	117	Down	9.37E‐08	Parkinson's disease
GO:0006103	20	Down	1.08E‐07	2‐oxoglutarate metabolic process
GO:0006120	41	Down	3.95E‐07	Mitochondrial electron transport. NADH to ubiquinone

**TABLE 5:** Top 20 genes associated with smoking

Entrez ID	Gene symbol	Fold change	Adj. *p* value
1545	CYP1B1	0.4135	<.001
1543	CYP1A1	0.8881	<.001
9002	F2RL3	0.1422	<.001
54360	CYTL1	0.2314	5.00 E‐04
130733	TMEM178A	0.2648	7.00 E‐04
23166	STAB1	0.1677	.0147
54492	NEURL1B	0.1658	.0303
6678	SPARC	−0.2189	.0306
80153	EDC3	0.0662	.0306
3992	FADS1	0.3898	.0998
4689	NCF4	0.1332	.1226
84215	ZNF541	−0.1279	.1226
26011	TENM4	−0.1598	.2758
23743	BHMT2	−0.1358	.4033
10494	STK25	0.0674	.4876
10243	GPHN	0.0762	.5127
158800	RHOXF1	−0.0662	.5127
10003	NAALAD2	0.1332	.5164
5604	MAP2K1	−0.1012	.5164
3421	IDH3G	0.0645	.5164

**TABLE 6:** Top 20 gene sets associated with smoking

Pathway ID	Number of genes	Direction	Adj. *p* value	Category name
hsa00280	44	Up	<0.001	Valine. leucine and isoleucine degradation
GO:0009404	12	Up	<0.001	Toxin metabolic process
GO:0050665	12	Up	9.00 E‐04	Hydrogen peroxide biosynthetic process
hsa00640	32	Up	0.0016	Propanoate metabolism
hsa00380	42	Up	0.0016	Tryptophan metabolism
GO:0009083	24	Up	0.0016	Branched‐chain amino acid catabolic process
GO:0006084	32	Up	0.0022	Acetyl‐CoA metabolic process
GO:0035383	91	Up	0.0043	Thioester metabolic process
GO:0009081	27	Up	0.0043	Branched‐chain amino acid metabolic process
GO:0006637	91	Up	0.0043	Acyl‐CoA metabolic process
GO:0006635	69	Up	0.0089	Fatty acid beta‐oxidation
hsa00071	43	Up	0.0148	Fatty acid degradation
hsa00020	30	Up	0.0148	Citrate cycle (TCA cycle)
GO:0009062	86	Up	0.0148	Fatty acid catabolic process
GO:0046395	227	Up	0.0248	Carboxylic acid catabolic process
GO:0016054	227	Up	0.0248	Organic acid catabolic process
GO:0019395	94	Up	0.0274	Fatty acid oxidation
hsa05310	30	Up	0.0287	Asthma
GO:0034440	96	Up	0.0306	Lipid oxidation
GO:0006085	18	Up	0.0787	Acetyl‐CoA biosynthetic process

**TABLE 7:** Top 20 genes associated with alcohol consumption

Entrez ID	Gene symbol	Fold change	Adj. *p* value
158056	MAMDC4	0.0664	.0043
122961	ISCA2	0.0775	.0043
284069	FAM171A2	0.0445	.0208
144233	BCDIN3D	0.0845	.0208
389203	SMIM20	0.0636	.0244
6016	RIT1	0.082	.0284
55449	DHRS4‐AS1	0.1512	.0297
2038	EPB42	−0.1687	.0415
25972	UNC50	0.0911	.0428
128977	C22orf39	0.0621	.1285
554	AVPR2	0.0475	.1375
54496	PRMT7	0.0609	.1375
5162	PDHB	0.0842	.1542
5224	PGAM2	0.1471	.1565
51522	TMEM14C	0.0722	.1565
3654	IRAK1	−0.1114	.1746
23034	SAMD4A	−0.0528	.1746
283927	NUDT7	0.1107	.1792
128346	C1orf162	−0.2169	.1820
7345	UCHL1	−0.2757	.1898

**TABLE 8:** Top 20 gene sets associated with alcohol consumption

Pathway ID	Number of genes	Direction	Adj. *p* value	Category name
GO:0070126	86	Up	<.001	Mitochondrial translational termination
GO:0032543	117	Up	<.001	Mitochondrial translation
GO:0070125	84	Up	<.001	Mitochondrial translational elongation
GO:0070124	84	Up	<.001	Mitochondrial translational initiation
GO:0022904	126	Up	<.001	Respiratory electron transport chain
GO:0022900	128	Up	<.001	Electron transport chain
GO:0033108	73	Up	1.00 E‐04	Mitochondrial respiratory chain complex assembly
GO:0097031	56	Up	1.00 E‐04	Mitochondrial respiratory chain complex I biogenesis
GO:0032981	56	Up	1.00 E‐04	Mitochondrial respiratory chain complex I assembly
GO:0010257	56	Up	1.00 E‐04	NADH dehydrogenase complex assembly
GO:0045333	193	Up	1.00 E‐04	Cellular respiration
GO:0031163	18	Up	4.00 E‐04	Metallo‐sulfur cluster assembly
GO:0016226	18	Up	4.00 E‐04	Iron–sulfur cluster assembly
GO:0046487	25	Up	4.00 E‐04	Glyoxylate metabolic process
hsa00190	119	Up	5.00 E‐04	Oxidative phosphorylation
GO:0006119	84	Up	7.00 E‐04	Oxidative phosphorylation
GO:0015936	17	Up	.0011	Coenzyme A metabolic process
GO:0019395	94	Up	.0011	Fatty acid oxidation
GO:0006635	69	Up	.0011	Fatty acid beta‐oxidation
hsa05016	177	Up	.0015	Huntington's disease

**TABLE 9:** Top 20 genes associated with HT use

Entrez ID	Gene symbol	Fold change	Adj. *p* value
170261	ZCCHC12	0.2202	<.001
80343	SEL1L2	0.4523	5.00 E‐04
400120	SERTM1	0.1426	.2945
9423	NTN1	0.1640	.4350
25884	CHRDL2	0.1356	.4903
140730	RIMS4	0.0701	.6069
6424	SFRP4	0.6362	.6069
8974	P4HA2	0.1608	.6493
26585	GREM1	0.3627	.7361
89876	MAATS1	−0.0950	.7730
220963	SLC16A9	0.1590	.7730
51081	MRPS7	0.0950	.7768
25878	MXRA5	0.2552	.7909
5387	PMS2P3	−0.0826	.7926
9201	DCLK1	0.2094	.7926
9540	TP53I3	0.1139	.7962
1136	CHRNA3	0.0695	.7962
4973	OLR1	−0.1745	.8054
6774	STAT3	0.1131	.8054
170261	ZCCHC12	0.2202	.8054

Abbreviation: HT, hormone therapy.

**TABLE 10:** Top 20 gene sets associated with HT use

Pathway ID	Number of genes	Direction	Adj. *p* value	Category name
GO:0006614	108	Up	.4887	SRP‐dependent cotranslational protein targeting to membrane
GO:0006613	110	Up	.4887	Cotranslational protein targeting to membrane
GO:0045047	112	Up	.4887	Protein targeting to ER
GO:0045058	43	Down	.4887	T cell selection
GO:0050855	15	Down	.4887	Regulation of B cell receptor signaling pathway
GO:0006415	178	Up	.4887	Translational termination
GO:0006414	207	Up	.4887	Translational elongation
hsa00640	32	Up	.4887	Propanoate metabolism
hsa05012	117	Up	.4887	Parkinson's disease
hsa00280	44	Up	.4887	Valine. leucine and isoleucine degradation
hsa00190	119	Up	.4887	Oxidative phosphorylation
GO:0070125	84	Up	.4898	Mitochondrial translational elongation
GO:0001887	115	Up	.4977	Selenium compound metabolic process
GO:0050853	46	Down	.4977	B cell receptor signaling pathway
hsa05010	161	Up	.4977	Alzheimer's disease
GO:0042775	62	Up	.4977	Mitochondrial ATP synthesis coupled electron transport
GO:0097296	20	Down	.5193	Activation of cysteine‐type endopeptidase activity involved in apoptotic signaling pathway
GO:0022904	126	Up	.5193	Respiratory electron transport chain
GO:0022900	128	Up	.5193	Electron transport chain
GO:0072599	116	Up	.5548	Establishment of protein localization to endoplasmic reticulum

Abbreviation: HT, hormone therapy.

**TABLE 11:** Top 20 genes associated with parity, sorted by fold change

	Gene symbol	Fold change	Adj. *p* value
7018	TF	0.0704	.9997
4246	SCGB2A1	0.0676	.9997
26353	HSPB8	0.0534	.9997
8483	CILP	0.0534	.9997
1396	CRIP1	0.0471	.9997
5519	PPP2R1B	0.0471	.9997
51716	CES1P1	0.0428	.9997
4653	MYOC	0.0426	.9997
399888	FAM180B	0.0383	.9997
6289	SAA2	0.0325	.9997
5918	RARRES1	−0.0648	.9997
26585	GREM1	−0.0568	.9997
1545	CYP1B1	−0.0529	.9997
347733	TUBB2B	−0.0495	.9997
220	ALDH1A3	−0.0447	.9997
54360	CYTL1	−0.0351	.9997
972	CD74	−0.0309	.9997
29990	PILRB	−0.0305	.9997
5414	SEPTIN4	−0.0304	.9997
338328	GPIHBP1	−0.0303	.9997

*Note*: As the parity variable gave a uniform distribution of *p* values, we present the genes with the 10 highest and lowest fold changes.

**TABLE 12:** Top 20 gene sets associated with parity

Pathway ID	Number of genes	Direction	Adj. *p* value	Category name
GO:0070126	86	Up	.8550	Mitochondrial translational termination
GO:0070124	84	Up	.8550	Mitochondrial translational initiation
GO:0070125	84	Up	.8550	Mitochondrial translational elongation
hsa00280	44	Up	.8550	Valine. leucine and isoleucine degradation
hsa05144	51	Up	.9994	Malaria
GO:0030316	87	Up	.9994	Osteoclast differentiation
GO:2000482	19	Up	.9994	Regulation of interleukin‐8 secretion
GO:1903034	403	Down	.9994	Regulation of response to wounding
GO:0002544	24	Up	.9994	Chronic inflammatory response
GO:0042554	23	Up	.9994	Superoxide anion generation
GO:0050777	120	Up	.9994	Negative regulation of immune response
GO:0032700	12	Up	.9994	Negative regulation of interleukin‐17 production
GO:0046849	77	Up	.9994	Bone remodeling
GO:0002712	42	Down	.9994	Regulation of B cell mediated immunity
GO:0002889	41	Down	.9994	Regulation of immunoglobulin mediated immune response
GO:0048771	150	Down	.9994	Tissue remodeling
GO:0006959	180	Down	.9994	Humoral immune response
GO:2000641	16	Up	.9994	Regulation of early endosome to late endosome transport
GO:0045124	33	Up	.9994	Regulation of bone resorption
GO:0033005	17	Down	.9994	Positive regulation of mast cell activation

When comparing gene expression profiles from breast tissue biopsies from women with BMI of 30 and above to those with BMI below 30, we identified 1577 significantly differentially expressed genes (Top 20 genes in Table 3). The differentially expressed genes included three alcohol dehydrogenases. There were more than 600 differentially expressed gene sets from GO and Kyoto Encyclopedia of Genes and Genomes, the majority of which were up‐regulated in women with obesity (Top 20 pathways in Table 4). The up‐regulated gene sets were dominated by immune‐related processes, with both innate and adaptive immunology represented. The list of down‐regulated gene sets included processes related to aerobic oxidation, fatty acid metabolism, amino acid metabolism, and protein translation in the mitochondria, which were all present among the top 20 gene sets, when sorted by *p* value.

Ten genes and 19 gene sets were statistically associated with smoking (Table 5). The genes CYP1B1, CYP1A1, F2RL3, CYTL1, TMEM178A, STAB1, NEURL1B, and EDC3 were significantly upregulated, whereas SPARC was downregulated. All the significant pathways were upregulated in smokers (Table 6).

Nine genes were statistically associated with alcohol exposure (Table 7). Eight of these were upregulated (MAMDC4, ISCA2, FAM171A2, BCDIN3D, SMIM20, RIT1, DHRS4‐AS1, and UNC50) and one, EPB42, was downregulated. Pathway analysis revealed 80 alcohol‐associated gene sets, and the 30 downregulated gene sets were all related to immunological processes (Top 20 pathways in Table 8). The 50 upregulated pathways were related to aerobic oxidation and fatty acid metabolism, and these were all among the top 20 gene sets when sorted by *p* value.

Two genes (ZCCHC12 and SEL1L2) were associated with HT use, both upregulated, but no pathways were identified (Tables 9 and 10). Finally, when comparing parous versus non‐parous women, we found no associated genes or pathways at our chosen level of statistical significance (*p* < .05), Tables 11 and 12.

We carried out a sensitivity analysis combining two sources of exposure data for smoking and alcohol: the detailed, eight‐page questionnaire answered 0–20 years prior to the biopsy, and the two‐page questionnaire answered at the time of the biopsy. Being classified as a current smoker when combining data from the two time points was associated with the same top five genes as having smoked during the last week before the biopsy (data not shown). Being classified as a former smoker when combining data from the two timepoints was not associated with any differentially expressed genes (data not shown). Assessed in the eight‐page questionnaire, the median amount of alcohol consumed was 3.08 g/day, and only four participants reported consuming more than 20 g/day. Information on alcohol intake in g/day collected 0–20 years prior to the biopsy was not associated with any differentially expressed genes.

## 4 DISCUSSION

In this study, we explored the association of known risk factors for breast cancer with gene expression profiles in 311 biopsies from normal breast tissue. The number of associated genes and pathways were the highest for obesity, followed by smoking and alcohol. HT use versus non‐use, and parity were associated with negligible differences in gene expression. The expression profiles of the biopsies where most likely influenced by the balance of cell types presented in the biopsy, as expected from this heterogeneous sample type.

### 4.1 Obesity

BMI was the most influential risk factor in this gene expression study, with 1577 differentially expressed genes, and more than 600 differentially expressed gene sets. Overall, processes like immunology, estrogen metabolism and energy metabolism dominated the BMI‐related results. In a wide perspective, our results reflect current hypotheses related to the causal association between obesity and breast cancer risk.

Our results indicate that immune‐related processes are activated in the breast tissue of women with obesity. We identified five toll‐like receptor (TLR)‐related gene sets, 34 interleukin‐related gene sets (including IL‐1, ‐1β, ‐2, ‐6, ‐8, and ‐17), 12 interferon‐related gene sets (IFN‐α, ‐β, ‐γ), and three NF‐κB gene sets. All of the mentioned gene sets were up‐regulated. This finding is in line with established hypotheses on adipose tissue as a mediator for establishment of chronic inflammation, which is ultimately linked to increased risk of cancer.^20^ During obesity, macrophages, putatively of the M1 type, accumulate in the adipose tissue, serving as a rich source of cytokines.^20^, ^21^ In obese breast tissue, inflammatory foci with dead adipocytes circled by macrophages, have been observed.^22^, ^23^ In the breast tissue, macrophages are exposed to saturated fatty acids from lipolysis leading to TLR 4 signaling via NFκ‐b, culminating in increased expression of pro‐inflammatory genes like COX‐2, IL‐1β, IL‐6, and TNF‐α.^20^ These obesity‐linked pro‐inflammatory mediators may have local, pro‐neoplastic effects, but also contribute to diminished overall health in obesity. However, our study cannot distinguish between breast tissue transcriptomic patterns associated with local inflammation, and transcriptomic patterns associated with systemic, circulating inflammatory factors. Biologically, however, this distinction is somewhat artificial, as the local and systemic effects of obesity are closely interrelated.^20^

Estrogen receptor signaling, increased by obesity and inflammation, is a key contributor to increased risk of hormone‐positive breast cancer. In our data, five prostaglandin‐related gene sets were identified. Prostaglandin contributes to increased aromatase expression in breast tissue, which is the rate‐limiting enzyme in estrogen biosynthesis.^24^, ^25^ Hence, our results support the “obesity‐inflammation‐aromatase” axis^23^ that leads to elevated estrogen levels in obese, postmenopausal women, resulting in increased breast cancer risk. The down‐regulated gene sets in our results were dominated by processes such as aerobic oxidation and fatty acid metabolism. Imbalances in these energy metabolism pathways are closely linked to accumulation of body fat leading to obesity.^26^ In the breast, adipocytes are involved in normal tissue development, but there is also close interaction between stromal adipocytes and tumor cells.^27^ Our results are in line with the finding of gene expression related to lipogenesis and fatty acid oxidation being downregulated in subcutaneous fat of both moderately and morbidly obese women, potentially as a mechanism for limiting further development of fat mass.^28^, ^29^ Of note, it has been suggested that this profile may be reversed by tumor cells, allowing adipocytes to provide lipids for the growing tumor.^27^

We also identified several down‐regulated gene sets related to protein translation in the mitochondria. Metabolic imbalance is closely related to mitochondrial function, as they in addition to ATP production are involved in production and elimination of reactive oxygen species (ROS).^30^ Obesity causes increased inflammation and oxidative stress through ROS production, which in turn may lead to mitochondrial dysfunction.^30^ In adipose tissue and skeletal muscle, numbers of mitochondria and rate of mitochondria biogenesis may decrease during obesity.^30^ Although these processes have not been described in breast tissue, our gene expression data from normal breast tissue supports this overall concept.

### 4.2 Smoking

Our results revealed several genes and pathways significantly associated with smoking (Tables 5 and 6). Eight genes were up‐regulated, one gene was down‐regulated, and 19 gene sets were associated with smoking. Cytochrome P‐450 1A1 and ‐1B1 (CYP1A1 and CYP1B1) were among the top up‐regulated genes. These genes are involved in metabolism of carcinogens including combustion products like polycyclic aromatic hydrocarbons found in cigarette smoke, but they are also involved in estrogen metabolism,^31^ as well as breast cancer proliferation and survival.^32^ Several aspects of CYP gene biology (expression levels, methylation levels, gene function) are related to smoking. Tsai et al.^33^ investigated smoking‐associated DNA methylation and gene expression variation in adipose tissue biopsies. Five of the identified genes in that study (AHRR, CYP1A1, CYP1B1, CYTL1, F2RL3) were both hypo‐methylated and upregulated in current smokers. Four of those five genes were identified in our study (CYP1A1, CYP1B1, CYTL1, and F2RL3). Furthermore, CYP gene biology was associated with smoking in studies of breast cancer patient survival,^34^ lung tissue,^35^, ^36^ prostate cancer cells,^37^ and fetal placenta and livers.^38^ Complementing these previous findings with our smoking‐related gene expression data in normal breast tissue, gives important clues to the biological effects of smoking, that may contribute to increased breast cancer risk.

Similar to our findings on CYP1A1 and –B1, coagulation factor II receptor‐like 3 (F2RL3, coding for the proteinase‐activated receptor 4 protein) was up‐regulated in our data, in accordance with previous reports. In an epigenome‐wide study of DNA from pre‐diagnostic blood samples, F2RL3 hypo‐methylation strongly correlated with smoking.^39^ Further, F2RL3 methylation was suggested as a biomarker of smoking,^40^ and over‐expression and hypo‐methylation were associated with higher risk of lung cancer,^41^ and with tumor aggressiveness and poor survival in renal cancer.^42^

In the smoking group, stabilin‐1 (STAB1) was up‐regulated, and secreted protein acidic and rich in cysteine (SPARC) was down‐regulated. STAB1 is a scavenger receptor mediating both phagocytosis of unwanted self‐components, intracellular sorting, and endocytosis of extracellular ligands such as the extracellular matrix component SPARC.^43^ STAB1 was up‐regulated in smokers and COPD patients compared to non‐smokers, although non‐significantly.^44^ STAB1 is expressed on tumor‐associated macrophages in several cancers, and in human breast cancer STAB1 was found in stage I and IV disease, suggesting a role in early primary tumor growth and progression.^43^ Studies have reported that SPARC induction inhibits breast cancer cell proliferation,^45^ and down‐regulated expression of SPARC correlated with poor breast cancer prognosis.^46^ However, the role of SPARC may be highly dependent on context.^47^

Among the 19 pathways associated with smoking, the most prominent feature were energy metabolism and fatty acid metabolism pathways, including fatty acid degradation processes, acetyl‐CoA metabolism, and the citrate cycle. Smoking has well‐established effects on adipose tissue, termed smoking‐induced dyslipidemia. In this state, lipolysis and free fatty acids are increased, involving hormone sensitive lipase and adipocyte differentiation.^48^ Systemically administered nicotine induces lipolysis, in part by activating the classical adrenergic mechanism, and in part by directly activating a nicotinic cholinergic lipolytic receptor located in adipose tissue.^49^

### 4.3 Alcohol

Exposure to alcohol was associated with 9 differentially expressed genes, and 80 gene sets (Tables 7 and 8). Overall, the magnitude of the differential gene expression is comparable to previous analyses of alcohol and gene expression in breast tumors.^50^ Interestingly, the up‐regulated BCDIN3 domain containing RNA methyltransferase gene (BCDIN3D) has been clearly linked to breast cancer progression, via down‐regulation of tumor suppressor miRNAs.^51^ In a cohort of 227 breast cancer patients, tumor levels of BCDIN3D was associated with lower disease‐free survival.^52^ Hence, BCDIN3D could serve as a link between alcohol consumption and breast cancer tumorigenesis and survival.

Among the 80 pathways associated with alcohol consumption, 30 were down‐regulated. All of these were related to immunological processes, and the majority describe aspects of the innate immune system. Particularly, mast cell mediated immunity was present, including mast cell activation and degranulation. Mast cells have been linked to alcohol consumption, as they may mediate the damaging effects of alcohol by contributing to chronic inflammation, tissue damage, and remodeling, especially in the gastrointestinal tract.^53^ Their role in cancer,^54^ including breast cancer, is controversial, with conflicting results on the association with disease subtypes and prognosis.^55^, ^56^ In sum, these findings warrant further investigation of the effects of alcohol in the pre‐cancerous breast tissue environment.

Fifty pathways were up‐regulated in alcohol consumers (Table 8). Among those, two related processes were represented: aerobic oxidation, including translation of mitochondrial proteins for oxidative phosphorylation, and fatty acid metabolism. As in the liver, alcohol is metabolized in breast tissue into acetaldehyde, a class 1 carcinogen forming DNA and protein adducts, and further into acetic acid and acetyl‐CoA, the latter which enters the citric acid cycle.^57^, ^58^ This increased acetyl‐CoA input may drive energy metabolism and increase the cellular energy state.^59^ Furthermore, it has been suggested that acetyl‐CoA is not merely a passive metabolite, but rather an important signaling molecule dictating cell function in a variety of settings.^58^ Similarly, the various metabolites of the tricarboxylic acid cycle (TCA), increasing in concentrations upon an up‐regulation of the cycle, affect intracellular and organismal processes, such as innate immunity, inflammation, and immune effector cells (succinate), and tumor cell growth (fumarate). TCA metabolite release from the mitochondria are one of the main processes by which the mitochondria influence cell function.^58^ With the upregulation of aerobic oxidation pathways in our data, perhaps as a response to increased levels of acetyl‐CoA from ethanol, it is also evident that the genotoxic acetaldehyde may be present in the breast tissue. Taken together, our data suggests that alcohol consumption may influence gene expression related to breast tissue physiology and metabolism.

### 4.4 Hormone therapy

A current exposure to HT in our study was associated with two upregulated single genes, but no significant pathways (Tables 9 and 10). Prolonged, systemic use of all, yet especially combined HT is associated with increased risk for breast cancer.^60^ Hall et al. found a distinct gene expression profile in breast cancer tissue associated with HT use, and linked HT use to better recurrence‐free survival.^61^ Changes in gene expression patterns in normal breast tissue after treatment with HT was observed in one experimental study,^62^ and a recent study on DNA methylation showed association between HT use and epigenetic changes in normal breast tissue.^63^ We did not observe similar changes in our data. Few participants exposed and lack of information on prior HT use, as well as duration of use, could partially explain these results.

### 4.5 Parity

In our study, parity was not associated with any significant single genes or pathways (Tables 11 and 12). Epidemiological studies have shown that a first full time pregnancy at an early age, as well as multiple pregnancies, are associated with long‐term risk reduction for breast cancer.^64^ Several studies found genomic signature of pregnancy in the breast tissue by comparing gene expression profiles of parous and non‐parous postmenopausal women.^65^, ^66^ We did not reproduce these findings, perhaps related to a low number of non‐parous women in our cohort (55 women, 17% of the study sample).

### 4.6 Sensitivity analysis

As a sensitivity analysis, we combined two sources of exposure data for smoking and alcohol: the detailed, eight‐page questionnaire answered 0–20 years prior to the biopsy, and the two‐page questionnaire answered at the time of the biopsy. The combined information on smoking exposure provided no further insight. This was also true for participants being classified as former smokers. Similarly, combining information on alcohol intake during the week before the biopsy with the data on alcohol intake 0–20 years prior to the biopsy provided no further insight. The average alcohol consumption of our participants was low (median: 3.08 g/day), which limits our ability to discern effects of higher alcohol consumption.

For these sensitivity analyses of smoking and alcohol, the detailed exposure information was separated with up to 20 years in time from the more limited two‐page questionnaire answered at the time of the biopsy. As the additional data did to add much, we chose to present the most recent exposure information as our main result, even though it was less detailed compared to the eight‐page questionnaire. The lack of additional findings ties in with the understanding of gene expression being a highly dynamic and responsive biological process, which is likely to reflect recent exposures rather than exposure history. The results are in line with findings on gene expression in blood related to smoking history and current smoking.^67^ In comparison, DNA methylation patterns may to a larger extent reflect previous exposure.^68^

## 5 STRENGTH AND LIMITATIONS

The main strength of this study is the analysis of normal breast tissue samples from cancer‐free women. With our choice of sample material, we were able to describe the variability of gene expression according to established breast cancer risk factors in women with no clinically detected breast abnormalities. Our relatively large sample size also improves the generalizability of the findings, compared to many other studies. Further, placing the biopsies directly in an RNA stabilizing agent after surgical removal diminishes the risk of ex vivo expression changes and RNA degradation during storage. Information on current exposures is important for gene expression profiling, and these were collected at the time of the biopsy sampling.

Several limitations must be considered. We collected whole biopsies, and no histological assessment of the tissue composition was performed. In general, using self‐report as the data collection method may introduce information bias, and unmeasured confounding factors may influence our findings. Twenty‐one percent of our study population were defined as smokers. This number is comparable to smoking prevalence for adult females in Norway in 2010, at the time of our sample collection.^69^ We did not assess smoking beyond current smoking status in our main analysis. Hence, a certain degree of misclassification is expected, for example, in categorizing former smokers as non‐smokers, which may drive our results toward the null. However, our sensitivity analysis supports that smoking history does not have effect on gene expression in the breast tissue. Further, alcohol consumption is associated with smoking and is itself a known risk factor for BC. We adjusted for alcohol intake in the smoking analyses. Nonetheless, statistical adjustment using self‐reported alcohol consumption may not be adequate to control fully for confounding by alcohol. Our alcohol‐related analysis also has a few limitations. There was a high percentage of alcohol consumers in this study, but the proportion is comparable to the whole NOWAC study.^70^ Additionally, we only have data on alcohol consumption during the previous week before biopsy in our main analysis. Potential effects of alcohol dose were addressed in the sensitivity analysis, although, these data were separated by up to several years from the biopsy. Finally, we did not include any analyses stratified by type and amount of alcohol, due to loss of power in such subgroup analyses.

The descriptive, cross‐sectional design of this study provides a snapshot in time of gene expression profiles and does not allow any discussion of causality. By its nature, gene expression analysis is hypothesis generating. Testing the identified gene expression associations by using other study designs such as randomized controlled trials, or in an experimental, in vitro setting was beyond the scope of the present study.

## 6 CONCLUSION

To our knowledge, this is the first study describing associations of breast cancer related exposures and gene expression profiles, in normal breast tissue from cancer‐free, post‐menopausal women. Obesity, smoking, and alcohol had the highest numbers of associated genes and pathways, whereas HT use and parity were associated with negligible gene expression differences in our data. Our results provide both confirmation of some previously reported findings, but also new hypotheses for further exploration. We conclude that our data provide an informative baseline for improved understanding of exposure‐related molecular events in normal breast tissue.

## AUTHOR CONTRIBUTIONS

**Sanda Krum‐Hansen:** Conceptualization (equal); data curation (supporting); formal analysis (supporting); investigation (lead); methodology (equal); writing – original draft (equal); writing – review and editing (equal). **Karina Standahl Olsen:** Supervision (supporting); visualization (supporting); writing – original draft (equal); writing – review and editing (equal). **Endre Anderssen:** Data curation (lead); formal analysis (lead); methodology (supporting); software (lead); visualization (lead); writing – original draft (supporting); writing – review and editing (supporting). **Jan Ole Frantzen:** Conceptualization (supporting); funding acquisition (supporting); investigation (supporting). **Eiliv Lund:** Funding acquisition (lead); project administration (lead); resources (equal); supervision (supporting). **Ruth H. Paulssen:** Conceptualization (equal); funding acquisition (supporting); methodology (equal); project administration (supporting); resources (equal); supervision (lead); writing – original draft (supporting); writing – review and editing (supporting).

## CONFLICT OF INTEREST

The authors have stated explicitly that there are no conflicts of interest in connection with this article.

## DISCLAIMER

The gene expression laboratory analyses were provided by the Genomics Core Facility (GCF), Norwegian University of Science and Technology (NTNU). GCF is funded by the Faculty of Medicine and Health Sciences at NTNU and Central Norway Regional Health Authority.

## ETHICS STATEMENT

The Regional Committee for Medical and Health research Ethics (case no # 200603551) has approved this study. Participants in this study signed an informed consent form.

## Supporting information


**Figure S1**: Results of PCA analysis illustrated with exposure variables.

**Table S1**: Results of cellular deconvolution.

**Table S2**: The list of all differentially expressed genes.

**Table S3**: The list of all differentially expressed gene set.

## Data Availability

Due to ethical restrictions on this dataset, which contains potentially sensitive information, the data will be made available upon request. Please contact the authors (karina.s.olsen@uit.no).
