# Supplementary figures and images for: Associations of breast cancer related exposures and gene expression profiles in normal breast tissue—The Norwegian Women and Cancer normal breast tissue study
Source: Cancer Rep (Hoboken). 2023 Jan 8;6(4):e1777. doi: 10.1002/cnr2.1777 (PMC10075301; doi:10.1002/cnr2.1777)

Alcohol

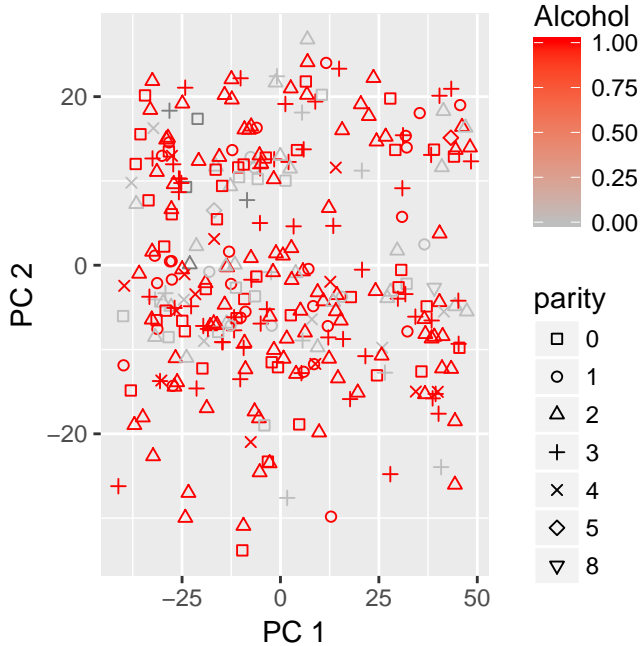

Smoking

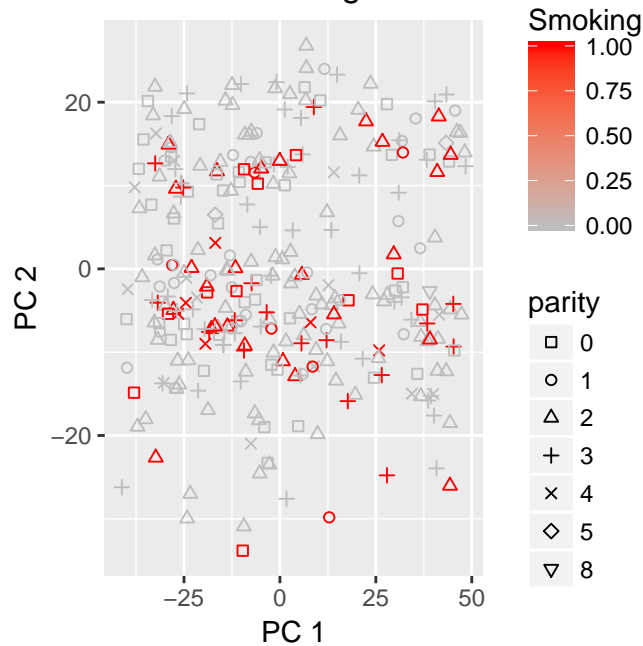

HRT

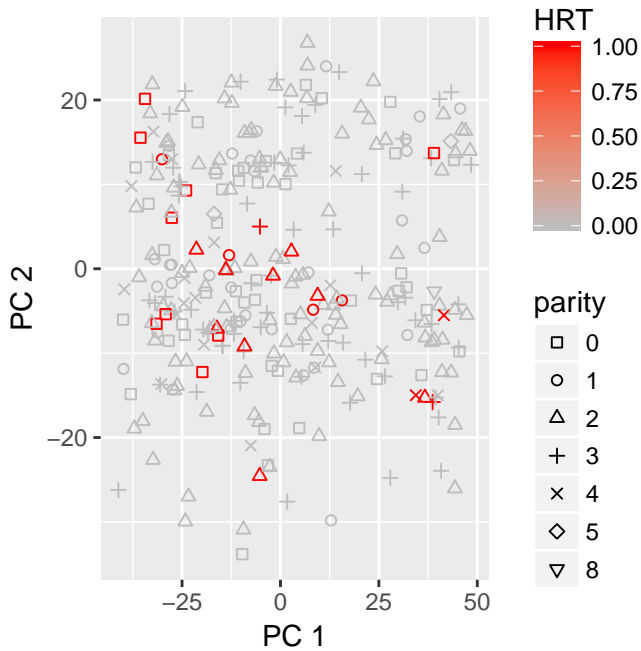

BMI

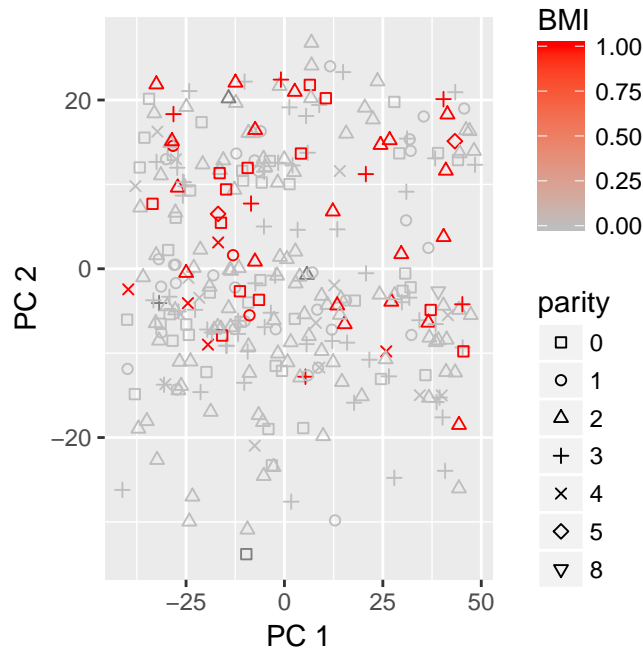

Supplement: Supplementary file 1 — Figure S1: Results of PCA analysis illustrated with exposure variables. [file CNR2-6-e1777-s003.pdf]
