# Supplementary material for: Associations of breast cancer related exposures and gene expression profiles in normal breast tissue—The Norwegian Women and Cancer normal breast tissue study
Source: Cancer Rep (Hoboken). 2023 Jan 8;6(4):e1777. doi: 10.1002/cnr2.1777 (PMC10075301; doi:10.1002/cnr2.1777)
Supplement: Supplementary file 2 — Table S1: Results of cellular deconvolution. [file CNR2-6-e1777-s001.docx]

|  |  | Cell deconvolution factors | | | Principal component scores | | |
| --- | --- | --- | --- | --- | --- | --- | --- |
| Marker gene | Cell type | NMF1 | NMF2 | NMF3 | PC1 | PC2 |  |
| CDH1 | Epithelial | 0,92 | -0,92 | -0,08 | -0,92 | 0,04 |  |
| EPCAM | Epithelial | 0,91 | -0,91 | -0,07 | -0,93 | 0,01 |  |
| LEP | Adipose | -0,75 | 0,72 | -0,04 | 0,84 | 0,05 |  |
| HOXC8 | Adipose | -0,63 | 0,82 | -0,12 | 0,89 | 0,15 |  |
| HIF1A | Lymph | -0,15 | -0,01 | 0,86 | -0,01 | -0,89 |  |
| IL10 | Lymph | 0,15 | -0,28 | 0,76 | -0,25 | -0,78 |  |
